# From Challenge to Change: The Journey of Millennial Nurse Unit Managers in a Shifting Healthcare Landscape

**DOI:** 10.1155/jonm/3602764

**Published:** 2025-10-25

**Authors:** Jiae Lee

**Affiliations:** Department of Nursing, Seojeong University, Yangju, Republic of Korea

**Keywords:** intergenerational relations, leadership, nurse administrator, nurse manager, qualitative research

## Abstract

**Background:**

As the baby boomer generation retires, millennials increasingly assume leadership roles in hospital organizations, including as nursing unit managers. Millennials are recognized for their focus on personal growth, digital skills, and flexible work preferences; however, they face unique challenges in managing healthcare teams. Therefore, this study aimed to explore the challenges millennial nurse unit managers face in unit operations and the strategies they use to overcome them.

**Methods:**

This qualitative study recruited 19 women participants, aged 36–43, from tertiary and general hospitals in Seoul, South Korea. Data were collected from 19 participants using semistructured interviews.

**Results:**

Content analysis identified four themes that nurse unit managers adopt to overcome their challenges: innovation in intergenerational communication, identification as a leader, adaptation to technological changes, and management of work–life integration.

**Conclusion:**

This study's findings provide valuable insights into millennial nursing unit managers' roles, leadership approaches, and necessary competencies. Based on the findings, strategies to enhance their competencies are suggested.

**Implications for Nursing Management:**

Nursing management should implement tailored training programs and organizational strategies to empower millennial nurse unit managers, thereby enhancing leadership effectiveness, workforce engagement, and healthcare quality and settings.

## 1. Introduction

The management tier in healthcare organizations has traditionally been composed of baby boomers (born 1946–1964) and Generation X (born 1965–1980) [1]. However, the retirement of baby boomers has led to increased recruitment and promotion of millennials (born 1981–1996), making them a demographic of significant influence in the workforce [1]. Millennials are the largest generational cohort with a population of 1.8 billion; therefore, they are expected to maintain dominance in the years ahead [2]. In contrast to previous generations, millennials prioritize careers that offer growth opportunities and are highly motivated by monetary rewards, reflecting a generational shift in workplace values [3, 4]. They prefer flexible workplace structure and demonstrate strong digital proficiency, actively engaging with emerging content through social media platforms [5]. Therefore, the healthcare field must recognize these unique traits and develop targeted support systems to enable millennial managers to thrive in managerial roles [6].

Nursing unit managers' competencies are crucial for hospital organizations to adapt to new challenges and achieve health policy goals [7]. Effective hospital management relies on a combination of “hard competencies,” such as specific technical knowledge and skills acquired through practical training, and “soft competencies,” including adaptability, leadership, effective communication, teamwork, time management, creativity, and decision-making [8]. Nursing unit managers are key leaders within healthcare settings and must possess and develop the required hard and soft competencies to navigate the complexities of modern healthcare and ensure organizational success [7, 9].

However, millennial nurse unit managers have been found to exhibit lower competence in enhancing nursing care quality compared to their Generation X counterparts [10] and to experience difficulties in building relationships with younger generations in terms of budgeting and clinical unit management [11]. While previous research has primarily emphasized the managerial difficulties experienced by millennial nurse managers [11], this study specifically focuses on nurse unit managers to provide deeper insights and develop targeted strategies for this pivotal managerial role.

There is a critical need to understand how millennial nurse unit managers successfully navigate the challenges of managerial roles and develop effective coping strategies. Therefore, this study aims to analyze the experiences of millennial nurse unit managers in overcoming managerial challenges and the strategies they utilized in the process.

## 2. Methods

### 2.1. Study Design

This qualitative research study employed the content analysis method proposed by Elo and Kyngäs [12]. Content analysis is a systematic and objective approach for describing and quantifying phenomena within textual data. It involves condensing raw data into concepts or categories that represent the studied phenomenon. This method focuses on identifying and organizing patterns and themes without necessarily aiming to develop new theories or explore lived experiences in depth [12]. This method was chosen as it is well suited to exploring and describing the managerial competence and challenges faced by nurse unit managers, allowing for a structured yet flexible analysis of interview data. Through this approach, data were systematically coded and categorized to develop a comprehensive understanding of the phenomenon.

### 2.2. Participants

The inclusion criterion was millennial generation individuals serving as managers of nursing units. Nineteen women aged between 36 and 43 years (mean age = 39.3) were recruited. A total of 19 participants completed the study without any withdrawals. Among them, 8 had completed a Master of Science in Nursing (MSN) and 11 held a Bachelor of Science in Nursing (BSN). Eight worked at a tertiary hospital, while the others worked at general hospitals. The participants' managerial experience ranged between 13 months and 7 years and 9 months, with an average of 3 years and 5 months.

### 2.3. Data Collection and Ethical Considerations

The study used purposive sampling and was conducted in Seoul, South Korea, between January 22, 2021, and August 31, 2021, after obtaining ethical approval from the institutional review board (ewha-202009-0020-01). The author initially contacted the nursing departments of eight hospitals that met the predetermined inclusion criterion of having more than 500 beds, encompassing both general and tertiary institutions. The study's purpose and methodology were clearly explained to the nursing department heads, and their cooperation was requested to facilitate participant recruitment. Subsequently, recruitment posters including contact information were distributed within these institutions to encourage voluntary participation by nurse unit managers. Nursing unit managers interested in participating in the study voluntarily were requested to contact the researcher. Once a nursing unit manager conveyed their willingness to participate via a phone call, the study's purpose and recording process were explained. Written consent was then obtained in person before starting the interview, at which point the date, time, and location were also confirmed. The interviews were conducted in a private setting chosen by the participant for their comfort, such as a meeting room or a nearby café, and lasted an average of 50 min per session. The interview questions were based on the study by Saifman and Sherman [13] and included the following: “What has your experience of becoming a nurse manager been?” “What is the biggest challenge in managing a nursing unit and why?” and “What kind of support do you think is necessary to enhance your competencies as a manager?” To clarify specific points from the initial interviews, follow-up phone interviews averaging 15 min were conducted with three participants. These interviews were recorded with consent, transcribed, and included in the analysis.

After 19 interviews, no new concepts emerged, indicating data saturation and no additional interviews were conducted. During the interviews, the participants' facial expressions, voice volume, and nonverbal cues were documented in field notes. The audio recordings of the interviews were subsequently transcribed into Korean for analysis.

### 2.4. Data Analysis

Data collection and analysis were carried out concurrently using content analysis [12]. Initially, the transcribed interview data were thoroughly reviewed to gain a deep understanding of the content. Next, a line-by-line analysis was conducted to identify significant units, including words or phrases that conveyed clear and consistent messages. These units were then categorized into higher-level units, including subthemes and overarching themes, through a series of discussions and consensus-building. The initial coding was conducted by the author using Covidence software. The author then repeated the coding process independently and compared the two sets of results. Field notes and reflective memos were recorded after each interview to monitor any shifts in interviewing style or researcher perspective. This process allowed the extraction of central themes that provided a holistic understanding of the challenges faced by millennial nursing unit managers and how they navigate them.

### 2.5. Rigor

The study's rigor was ensured by adhering to the criteria of credibility, transferability, dependability, and confirmability, as outlined by Lincoln and Guba [14]. For credibility, the author initially coded the data using Covidence software and later independently recoded the data after a time interval to minimize bias from prior interpretations. The two coding sets were then compared line by line. Any discrepancies were carefully reviewed by revisiting the original transcripts, cross-checking with field notes and memos, and refining code definitions when necessary to ensure consistency and analytical rigor. In addition, the transcribed interview data were validated with the participants, and the analytical findings were presented to two participants to confirm the accuracy of the interpretation. For transferability, interview data were collected until saturation was reached, ensuring that the findings accurately reflected the experiences of nursing unit managers. The interview data were meticulously collected, analyzed, and described to ensure dependability. To ensure dependability, the same primary interviewer conducted all interviews throughout the extended data collection period using a standardized semistructured guide. This approach helped maintain consistency in the way data were collected over time. Regarding enhancing confirmability, minimizing bias, and ensuring reflexivity, the recorded interviews were revisited to check for potential interviewer interventions or subjective judgments.

Specifically, the author is a female researcher with a clinical background and previous experience conducting qualitative interviews with staff nurses and nurse unit managers. The author, as a member of the millennial generation, empathizes with the unique challenges faced by this in-between generation, which sparked a genuine interest in exploring the research topic and helped build rapport with the study participants, thereby deepening the understanding of their experiences. To further ensure reflexivity and minimize researcher bias, the author engaged in bracketing by identifying and setting aside potential preconceptions formed through prior clinical experience, documenting them prior to data analysis. These included the assumptions that (1) nurse unit managers may prioritize organizational needs over those of staff nurses; (2) they may possess a broader perspective due to their extensive clinical experience; and (3) they may be more attuned to interpersonal conflict owing to their familiarity with hierarchical organizational culture. The researcher had no prior contact with the participants before data collection, and to provide an external perspective on the analysis, the findings were reviewed by two individuals who did not participate in the study: a nurse unit manager and a qualitative nursing researcher.

## 3. Results

The analysis of millennial nursing unit managers' experiences in overcoming challenges identified eight subthemes and four main themes (Table 1).

### 3.1. Innovation in Intergenerational Communication

The participants recruited were millennials, also known as the “sandwiched” generation, who face challenges in building relationships with older and younger generations. To strengthen communication with other generations, the millennial participants stated that they first identified the distinct approaches of each group and then devised generation-specific communication strategies.

#### 3.1.1. Effective Communication Strategies With Older Senior Managers

The analysis showed that the conservative older generation tended to dismiss the participants' opinions, disregarding their realistic and innovative ideas for running nursing units under the guise of preserving nursing traditions. The participants stated that they aimed to persuade the older generation by using evidence-based arguments and strategically presenting their opinions aligned with others' interests. For example, participants stated the following:

*I'm caught between upper management and new nurses. While I'm told to support the new staff, there's little consideration for younger managers like me. To address this, I use data—like bed occupancy rates and turnover figures—to communicate workload more objectively. (Participant 7)*

*New managers' opinions are often dismissed as inexperienced. I've realized that gaining empathy from senior leadership is key, since they tend to view things from their own perspective. (Participant 18)*

#### 3.1.2. Innovative Mentorship Strategies for Novice Nurses

The participants perceived a generational gap and faced difficulties in communicating and building relationships with the younger nurses in their units. The participants reported that the novice nurses were proactive in requesting work tasks; however, they maintained distance during one-on-one meetings. Additionally, participants perceived that the existing preceptor system contributed to conflicts. To address these issues, some participants stated that they had introduced a mentoring system between themselves and novice nurses, aiming to act as role models. Moreover, the participants described fostering a positive organizational culture by educating novice nurses on the importance of peer consideration and teamwork. Examples of the transcribed interviews included the following:

*I've noticed a generational gap. New nurses approach me directly for guidance but remain distant in personal interactions. To build stronger relationships, I replaced the senior nurse–new nurse mentoring system with one led by me, the unit manager, so I can be a role model. (Participant 13)*

*Tasks like changing sheets are much faster with teamwork, but new nurses often don't step in to help. I sensed a lack of consideration, which led to conflict under the traditional preceptor system. I decided to personally guide them on teamwork and mutual support. (Participant 17)*

### 3.2. Leader Identity

After becoming nurse unit managers, the participants reported experiencing identity confusion, as their roles included being a manager and staff nurse. Despite holding the same rank as senior managers, they did not receive peer recognition. Therefore, the participants described their efforts to find a balance between their roles and how they attempted to solidify their position within the organization.

#### 3.2.1. Balancing Nursing and Managerial Roles

Even after taking on the role of nurse unit manager, the participants reported they were still required to fulfill their roles as staff nurses; this created a dilemma due to the conflicting demands of nursing duties and management responsibilities. To address this, participants stated that they delegated tasks to charge nurses to ensure that the unit ran smoothly. Additionally, they attempted to balance their role expectations by striving to maintain an equilibrium between supporting on-site tasks and managing the unit. During heavy workloads, they provided direct ground assistance. Two participants stated their strategies to balance nursing and managerial roles as follows:

*As a nurse unit manager, I'm responsible for leading the unit but am still counted as a staff nurse. This dual role creates a dilemma, especially when I have to leave the unit for central training. To manage this, I delegate my clinical duties to the charge nurse to ensure continuity in patient care. (Participant 19)*

*It's not easy balancing both managerial and staff nurse roles. Still, I see value in being able to step in and support the team directly when needed. I try to maintain a balance between providing leadership and being present in the clinical field. (Participant 6)*

#### 3.2.2. Peer Recognition by Senior Managers

Despite becoming nurse unit managers, the participants were not recognized as peers by other previously appointed senior managers. In turn, this created an additional workload, which prompted the participants to strive for equal treatment as managers within the organization. During the interviews, participants communicated their strategies to overcome the hierarchical structure and their perceptions of how their peers viewed them. In addition, participants reported challenges in growing as managers resulting from unequal treatment from senior nurse unit managers; their strategies to overcome this included engaging in problem-solving and finding solutions together with their senior managers to assert their roles. Additionally, the patient assignment process was identified as a challenge; to overcome this, the unit managers explained departmental characteristics and situations to upper management to prevent unfair patient assignment practices and ensure equal standing, thereby avoiding any negative impact on the nurses. Two participants responded:

*The nurse unit manager who was in charge when I joined as a novice is still in the same role. Although we share the same title now, I'm not treated as an equal, which has hindered my growth. Still, I try to position myself as a collaborative colleague, not just someone following orders. (Participant 9)*

*As the youngest nurse unit manager, I've faced unfair treatment, such as being assigned more challenging patients. Despite having the same title, I'm not seen as an equal. I addressed this with upper management, explaining the specific needs of my unit, and asked for fairer treatment. I hope to establish myself as a respected and equal member among the managers. (Participant 4)*

### 3.3. Adaptation to Technological Changes

The participants perceived themselves as individuals who actively sought technological changes within the organization and, in this process, obtain the cooperation of nurses; however, securing sufficient technical support was a significant challenge. To overcome these difficulties, the participants reported leveraging leadership skills and creative ideas.

#### 3.3.1. Change Resistance Management

Familiar practices are comfortable and the participants reported encountering change resistance. For example, when they suggested technological changes, such as remote handovers and electronic nursing records, to better lead their units, they had to persuade the unit's nurses why these changes were necessary. The participants disclosed that they motivated nurses to adapt to the new technologies through education, workshops, incentives, and praise. For example, participants stated the following:

*When I introduced the electronic chart system, older nurses who were used to paper charts expressed fear and resistance, asking, ‘Why do we need to change?' To ease the transition, I provided ongoing education and gradually implemented the system, starting with basic features. Over time, they became more comfortable with the new system. (Participant 3)*

*The non-face-to-face handover system was met with skepticism—nurses doubted whether essential information could be shared without in-person interaction. Early issues like missing or duplicate information caused frustration. To encourage adoption, I emphasized the system's benefits and motivated staff by offering praise and small rewards. (Participant 5)*

#### 3.3.2. Technical Support and Resource Optimization

After the introduction of new systems, some participants perceived that work disruptions occurred due to a lack of technical support and training, prompting them to take various measures to resolve these issues. For example, the nurses' schedules and limited training time led to the creation of online materials and straightforward guidelines to efficiently answer frequently asked questions. Additionally, they worked closely with the technical department to establish a system for receiving prompt support in case of technical problems. Therefore, the nurses adapted to the new system, alleviating interdepartmental conflicts. Participants' responses included the following:

*After introducing the new system, frequent technical issues and insufficient training created challenges. Shift work made it hard for nurses to receive proper instruction, so I suggested creating online materials and simple guides to address common problems. (Participant 11)*

*Timely technical support and ongoing education are critical. Initially, delays in resolving system errors caused frustration and inter-departmental conflict. I facilitated meetings with the technical department to ensure collaboration, improving support so nurses had what they needed to adapt. (Participant 1)*

### 3.4. Management of Work–Life Integration

The participants disclosed efforts to achieve work–life harmony during the interviews. Being from the millennial generation meant that many participants had young children and family responsibilities. Their strategy to achieve a work–life balance included delegating appropriate tasks to nurses in their units. Additionally, participants reported striving to carry out their tasks efficiently within the limited working hours, aiming to avoid wasting time.

#### 3.4.1. Delegation of Nursing Tasks

Several participants reported introducing a committee system to classify the nature of tasks to improve their unit management's efficiency and the delegation of certain responsibilities to the units' nurses. Moreover, some reported appointing subleaders to ensure that the unit operated efficiently in their absence. This approach alleviated the unit managers' workload and created opportunities for nurses to showcase their capabilities. During the interviews, the participants made statements such as

*Balancing work and family has been challenging, especially with my young child and the many tasks in nursing unit management. To ease this, I established committees to delegate tasks like inventory, research, and quality improvement, allowing me to focus on core duties. (Participant 8)*

*Managing the unit during my absence was difficult, so I appointed sub-leaders for each shift to ensure smooth operations. This clarified task division and helped the sub-leaders grow, enabling me to focus on key decisions. (Participant 10)*

#### 3.4.2. Time Management–Focused Work

The participants reported taking proactive steps to utilize working hours efficiently by introducing a “no overtime policy.” This included streamlining and reducing unnecessary meetings and restructuring work processes as follows:

*The “no overtime policy” brought a meaningful change. I initiated it to improve work-life balance and led efforts to streamline workflows. We simplified nursing records, reduced repetitive reporting, and limited meetings to essential ones. These changes helped us complete tasks within work hours. (Participant 12)*

These changes supported nurses in completing their tasks within the designated time. To promote punctuality, a participant reported assigning heavy workloads to the unit's nurses and encouraged a culture of understanding and consideration among team members, enabling a work environment conducive to punctual arrivals and departures:

*Our unit struggled with both overtime and nurses arriving too early due to heavy workloads on junior staff. To address this, I promoted a supportive team culture and emphasized shared responsibility. By redistributing tasks more evenly, we worked toward an on-time work environment. (Participant 2)*

## 4. Discussion

Korean workplaces have traditionally been influenced by Confucian values emphasizing hierarchy, loyalty, and seniority, which shape interpersonal dynamics and leadership expectations [15]. These cultural norms often result in top-down communication and deferential attitudes toward senior managers [15]. However, the emergence of the millennial generation—with their emphasis on fairness, transparency, and self-actualization—challenges these traditional practices, leading to generational tensions and shifts in leadership styles [16]. Understanding this cultural context is crucial to interpreting the experiences of millennial nurse unit managers navigating these conflicting expectations and redefining organizational culture [17]. Reflecting this shift, participants acted as organizational culture change agents by seeking to assert themselves to the older generation and establish their position as equal peer managers. The millennial nurse unit managers in this study exemplified younger individuals' strong value on fairness in task assignments and promotions [18]. They used evidence-based arguments and strategically framed their ideas to align with others' interests. In doing so, they worked to demonstrate their equal roles and ensure fair patient assignments, thereby preventing negative impacts on the unit's team. These findings are consistent with those of Deal and Levenson [19], who noted that millennials are highly motivated by opportunities to share their ideas and pursue personal and professional growth, rather than performing repetitive and monotonous tasks. Furthermore, it is important to recognize that the generational characteristics of millennials may also create tension in traditionally hierarchical environments, as the participants in the study often experience difficulties when their input is disregarded within the rigid, feudal-like organizational culture. Unlike older generations who view loyalty to the organization as a virtue, millennials set high expectations for themselves, have a strong desire to outperform others, and value social recognition [17]. Since these characteristics can sometimes lead to conflicts with older generations, it is important to educate nursing unit managers about generational differences [20].

As supportive leaders, the participants also made efforts to build relationships and collaborate with the younger generation, namely, staff nurses, by introducing mentoring systems and promoting a positive organizational culture. To support adaptation to technological changes, they encouraged nurses through educational initiatives, workshops, and incentive programs. This finding aligns with those of a previous study by Nilsen et al. [21], who found that nurses can resist technological changes to the point of considering resignation to avoid accepting them. Building on this, involving nurses early in the change process, ensuring clear communication, and providing sufficient preparation time—along with helping them understand the purpose and benefits of the change for both patients and themselves—are essential strategies to enhance their willingness to accept and adapt to new technologies [22]. Therefore, it is important to provide ongoing education and training for nurse unit managers that focuses not only on interpersonal skills—such as critical thinking, team management, conflict resolution, and collaborative decision-making [23, 24]—but also on digital skills and adaptability to dynamic work situations [25]. Since these essential management competencies enable nurse unit managers to support nurses' job satisfaction and organizational commitment [23, 26], they highlight the importance of encouraging the comprehensive development of nurse unit managers [27].

Additionally, the participants described how they tackled technical training challenges, including providing online resources, guidelines, and prompt technical support; these interventions ultimately helped nurses to adapt to new systems and reduced interdepartmental conflicts. Introducing new technologies, such as applications, without proper training and relying solely on manuals has been shown to limit the effective acquisition of technologies [28]. Moreover, nurses and nurse unit managers may face difficulties resulting from a lack of proficiency, emphasizing the need for adequate educational support [28]. Furthermore, a study by Brown et al. [29] identified that nurses find new technologies uncomfortable because they are time-consuming; this reduces their direct patient care time and decreases work efficiency. Therefore, nurse unit managers must actively participate in designing and evaluating point-of-care technologies to effectively address nurses' challenges and promote smoother adoption and broader acceptance during implementation [29].

The participants in this study consciously tried to balance their dual roles, as nurse unit managers often face a dilemma between fulfilling managerial responsibilities and performing staff nurse tasks. This role conflict arises from the misalignment between these two identities, leading to conflicting goals when both are internalized simultaneously [30]. To manage this, participants delegated tasks to charge nurses and provided direct support during peak periods, illustrating practical strategies to navigate these overlapping roles. Vough et al. [30] argue that this conflict is best addressed by clearly distinguishing between roles. In hospital settings, middle-line managers frequently perceive themselves as being in a “management in training” phase, which contributes to a vague sense of identity during the transition to a managerial position [31]. However, research has shown that nurses' clinical experience enables them to better understand organizational dynamics and manage staff effectively, thereby facilitating a smoother and more positive shift from clinical to managerial roles [32]. Among Katz's three core managerial competencies—technical, human, and conceptual skills—the most critical for nurse unit managers is clinical skills, which refers to specialized knowledge and expertise in their specific role. This underscores the value of clinical competency in effective nurse leadership [33]. This highlights the importance of strengthening managerial competencies based on clinical experience. Managerial development programs should be structured to build upon and enhance these clinical foundations.

Participants emphasized the importance of achieving work–life balance amid the demanding nature of nurse unit management. To this end, some implemented committee systems and appointed subleaders to support unit operations. This not only alleviated their own workload but also created opportunities for staff nurses to demonstrate their capabilities. Given that nurse unit managers often work long hours, such initiatives are especially significant, as workload balance strongly correlates with job satisfaction among nurse managers [7, 33]. This focus on work–life balance reflects a broader societal and generational shift in South Korea. While past decades saw a dominant cultural emphasis on economic success often at the expense of personal time and self-actualization [16], there is now growing recognition of the need to balance career achievement with personal well-being [29]. Millennials in particular tend to prefer a work-to-leisure ratio of approximately 6:4, reflecting their desire for both professional success and a fulfilling personal life [16]. Building on these findings, it is crucial for nurse unit managers to establish clear and reasonable delegation standards based on task priority and complexity, while also implementing systems to continuously monitor and manage individual nurses' workloads [34]. Developing a more systematic management support framework will enhance operational efficiency and promote a sustainable work environment in nursing units.

The participants described implementing a “no overtime policy” and restructuring work processes to improve efficiency, promote an on-time work culture, and support nurses to balance workloads and maintain punctuality. Adhering to working hours and time management are key factors influencing job satisfaction [35]. Furthermore, clear delegation criteria and manageable workloads are essential for improving job satisfaction, which underscores the importance of nurse unit managers' competencies in effectively organizing tasks and reducing workplace stress [36]. Moreover, nurse unit managers should play an active role in creating positive environmental changes, as research shows that nurse empowerment is linked to leadership and decision-making abilities [27]. This suggests the need to strengthen leadership and decision-making training so that nurse unit managers can move beyond the role of mere middle managers and take an active role in fostering a positive organizational culture and leading change.

Overall, the millennial nursing unit managers interviewed in this study identified their challenges in unit operations and how they overcame them. The strategies they employed included recognizing and accommodating generational differences to improve leadership and communication, balancing their dual roles within the organization, implementing technological changes with the nurse's cooperation and organizational technical support, and delegating tasks to maintain a work–life balance while ensuring efficient use of working hours. Building on these findings, the participants emphasized leadership practices centered on soft competencies such as communication, teamwork, mentoring, and emotional support [8]. In particular, they actively applied these competencies to mediate generational conflicts within the organization and support the growth of junior nurses. These results align with previous research indicating that millennials perceive their leadership style as a “competency development–focused coaching” approach, characterized by actively supporting subordinates' learning and growth, encouraging participation in job-related training, and providing continuous feedback to foster competency development [1]. The influx of the millennial generation has brought about a shift in leadership styles, positively transforming organizational culture through soft competencies such as mentorship, communication, and adaptability [11], highlighting the need for tailored leadership development programs that reflect these changes.

### 4.1. Strengths and Limitations

This study is limited to female participants residing in Seoul, which may introduce selection bias and limit the generalizability of the findings. The extended data collection period may have introduced variability in organizational contexts, potentially influencing participants' experiences and perceptions. Nevertheless, this extended period also enabled capturing diverse experiences over time. Moreover, efforts to maintain consistent inclusion criteria and monitor organizational changes helped minimize potential contextual variability. Future research should include more diverse populations from different regions and gender groups to enhance the applicability of the results. Additionally, the study provides a comprehensive analysis of the challenges faced by millennial nursing unit managers, offering valuable insights into their leadership roles. The study's findings highlight key themes relevant to healthcare management, including intergenerational communication, leader identity, technological adaptation, and work–life balance. These findings offer practical recommendations to support the competency development of millennial nursing unit managers, which can enhance their professional practice.

## 5. Conclusions

This study explored the challenges faced by millennial nursing unit managers and the strategies they employed to address them. The findings suggest that competency development programs for this group should comprehensively focus on intergenerational communication, technological adaptation, role management and delegation, work efficiency, leadership for organizational change, mentoring and team collaboration, work–life balance, and clinical competency–based management. This study provides a comprehensive understanding of millennial nurse managers' perspectives amid ongoing generational shifts in the workplace. Future research should prioritize the design and evaluation of targeted educational interventions and include more diverse populations to enhance the generalizability of the findings.

## Figures and Tables

**Table 1:** Overview of the subthemes and main themes.

Subthemes	Main themes
• Effective communication strategies with older senior managers	Innovation in intergenerational communication
• Innovative mentorship strategies for novice nurses	

• Balancing nursing and managerial roles	Leader identity
• Peer recognition by senior managers	

• Change resistance management	Adaptation to technological changes
• Technical support and resource optimization	

• Delegation of nursing tasks	Management of work–life integration
• Time management–focused work	

## Data Availability

The supporting data are not available.
